# Reply to Sangster, G.; Luksenburg, J.A. Contamination of Consensus Sequences in Next-Generation Mitogenomics: The Published Mitochondrial Genome of *Haliastur indus* Is a Chimera with DNA from *Butastur indicus*. Comment on “Sonongbua et al. Insights into Mitochondrial Rearrangements and Selection in Accipitrid Mitogenomes, with New Data on *Haliastur indus* and *Accipiter badius poliopsis*. *Genes* 2024, *15*, 1439”

**DOI:** 10.3390/genes17010088

**Published:** 2026-01-14

**Authors:** Jumaporn Sonongbua, Worapong Singchat, Artem Lisachov, Kornsorn Srikulnath

**Affiliations:** 1 Animal Genomics and Bioresource Research Unit (AGB Research Unit), Faculty of Science, Kasetsart University, Bangkok 10900, Thailand; jumaporn.s@ku.th (J.S.); worapong.si@ku.th (W.S.); aplisachev@gmail.com (A.L.); 2 Interdisciplinary Graduate Program in Bioscience, Faculty of Science, Kasetsart University, Bangkok 10900, Thailand; 3 Faculty of Interdisciplinary Studies, Khon Kaen University, Nong Khai Campus, Nong Khai 43000, Thailand; 4 Special Research Unit for Wildlife Genomics (SRUWG), Department of Forest Biology, Faculty of Forestry, Kasetsart University, Bangkok 10900, Thailand; 5 Laboratory of Animal Cytogenetics and Comparative Genomics (ACCG), Department of Genetics, Faculty of Science, Kasetsart University, Bangkok 10900, Thailand; 6 Biodiversity Center Kasetsart University (BDCKU), Kasetsart University, Bangkok 10900, Thailand

We thank Dr. George Sangster and Dr. Jolanda A. Luksenburg for their careful assessment concerning the mitochondrial genome sequence of *Haliastur indus* (GenBank accession number: OP133375.1) [1], published in our article entitled “Insights into Mitochondrial Rearrangements and Selection in Accipitrid Mitogenomes, with New Data on *Haliastur indus* and *Accipiter badius poliopsis*” [2]. We appreciate the opportunity to respond and provide clarification.

After thorough re-examination, we identified an error during the reference-based assembly process. In the original de novo assembly, two scaffolds were obtained, but only one derived from mitochondrial DNA. When these scaffolds were merged, the mitogenome of the grey-faced buzzard (*Butastur indicus*, GenBank: AB830616.1, identical to the NCBI Reference Sequence NC_032362.1) was used as a reference. Consequently, the reference-based assembly erroneously incorporated a sequence from the *B. indicus* reference mitogenome, resulting in a chimeric mitogenome. Specifically, 10,078 bp of OP133375.1 originated from *H. indus* (corresponding to positions 1674–11,751), while the remaining 8977 bp were derived from the *B. indicus* (positions 1–1673 and 11,752–19,055 of OP133375.1), leading to the erroneous inclusion of mitochondrial genes (e.g., *ND5*, *Cytb*, *ND6*, both rRNAs, and eight tRNAs, including *tRNA-Phe*, *tRNA-Val*, *tRNA-Ser*, *tRNA-Leu*, *tRNA-Thr*, *tRNA-Pro*, and *tRNA-Glu*) from the latter species.

To correct this issue, we resequenced the same sample using 100 ng/μL DNA on an Illumina NovaSeq™ 6000 platform (2 × 150 bp paired-end, 15× coverage) and combined the new reads with previous data to increase depth. De novo assembly with GetOrganelle (v1.7.4.1) [3] produced a 17,968 bp backbone containing 13 protein-coding genes, rRNAs, tRNAs, and partial control regions. To resolve the repeat-rich control regions, additional PCR amplification and sequencing were performed using Sanger sequencing on an Applied Biosystems 3730xl DNA Analyzer (Thermo Fisher Scientific, Waltham, MA, USA) and long-read sequencing on a PromethION P2i (Oxford Nanopore Technologies, Oxford, UK). All fragments from Illumina, Sanger, and Nanopore data were merged in Geneious Assembler (v2025.0.3), yielding a 19,986 bp circular mitogenome with no ambiguous bases or structural gaps.

Gene annotation identified the standard avian mitochondrial gene complement, comprising 13 protein-coding genes, 22 tRNAs, 2 rRNAs, and duplicated control regions, as previously reported in Accipitriformes [4]. Read-mapping analyses confirmed uniform coverage across the corrected assembly (mean coverage 96x; median 93x), with reduced depth within the pseudo-control region. This low-coverage segment, caused by tandem repeats that impede short-read mapping, was resolved by incorporating long-read Nanopore data, confirming the completeness and reliability of the final assembly. Alignment of the corrected *H. indus* mitogenome (OP133375.2) against the previous chimeric version (OP133375.1) and the *B. indicus* reference (NC_032362.1) confirmed complete removal of the *B. indicus*-derived fragment (Figure 1). Pairwise identity analysis further supported this correction: OP133375.1 showed 93.9% identity with *B. indicus*, whereas OP133375.2 exhibited lower identity (80.5%) with *B. indicus*, consistent with successful removal of the chimeric fragment. Comparative phylogenetic analyses were conducted using the maximum likelihood (ML) method with 1000 ultrafast bootstrap replicates, implemented in the IQ-TREE web server [5], based on 13 protein-coding genes and two rRNAs from 24 Accipitridae species, with *Pandion haliaetus* (Pandionidae) designated as the outgroup. All genes placed *H. indus* mitogenome (OP133375.2) in the same clade as *Milvus migrans*, consistent with the true phylogenetic position of *H. indus* (Figure 2) [6].

In summary, our re-examination and reassembly confirm that the corrected *H. indus* mitogenome (OP133375.2) represents the authentic mitochondrial genome of *H. indus*. The updated sequence has been submitted to GenBank and will be accompanied by a correction publication. We sincerely appreciate the constructive feedback, which has helped improve the accuracy and clarity of our study.

## Figures and Tables

**Figure 1:**
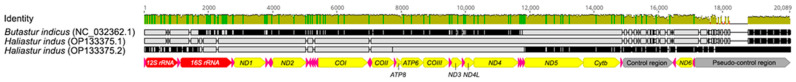
Sequence alignment showing that the *Butastur indicus*-derived fragment present in the previous chimeric version (OP133375.1) was removed in the corrected *Haliastur indus* mitogenome (OP133375.2). Black bars indicate sequence differences, while grey shading denotes positions where the sequences are identical. Colored arrows indicate annotated genomic features: yellow, protein-coding genes; red, rRNAs; pink, tRNAs; and grey arrows indicate control and pseudo-control regions.

**Figure 2:**
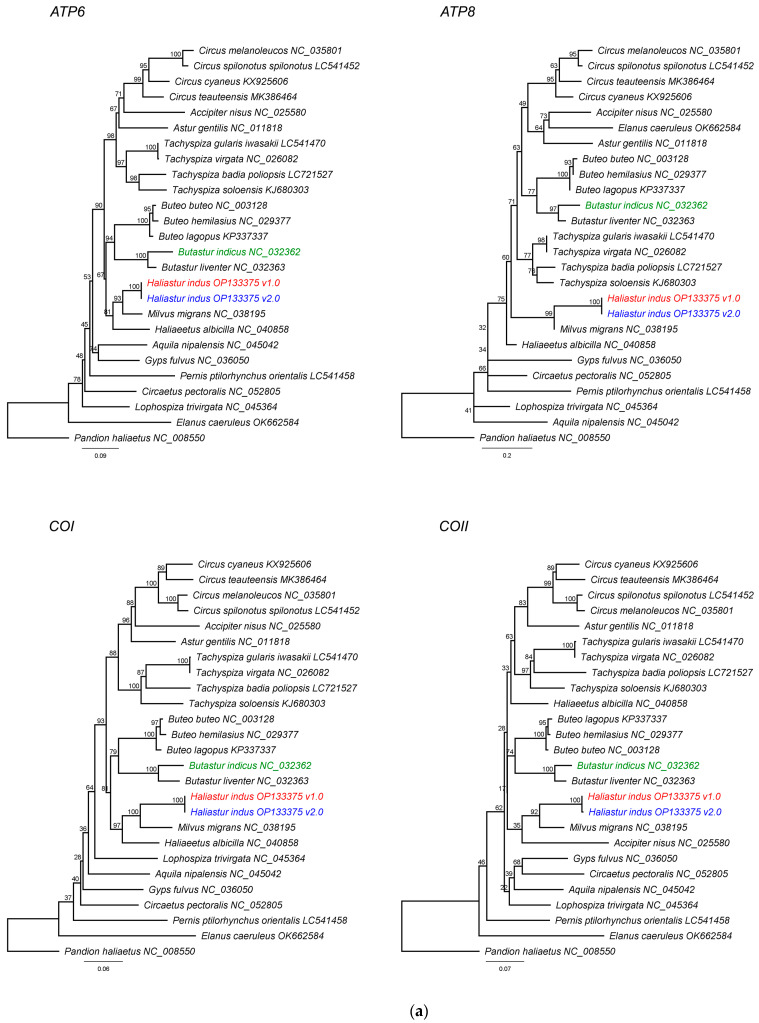

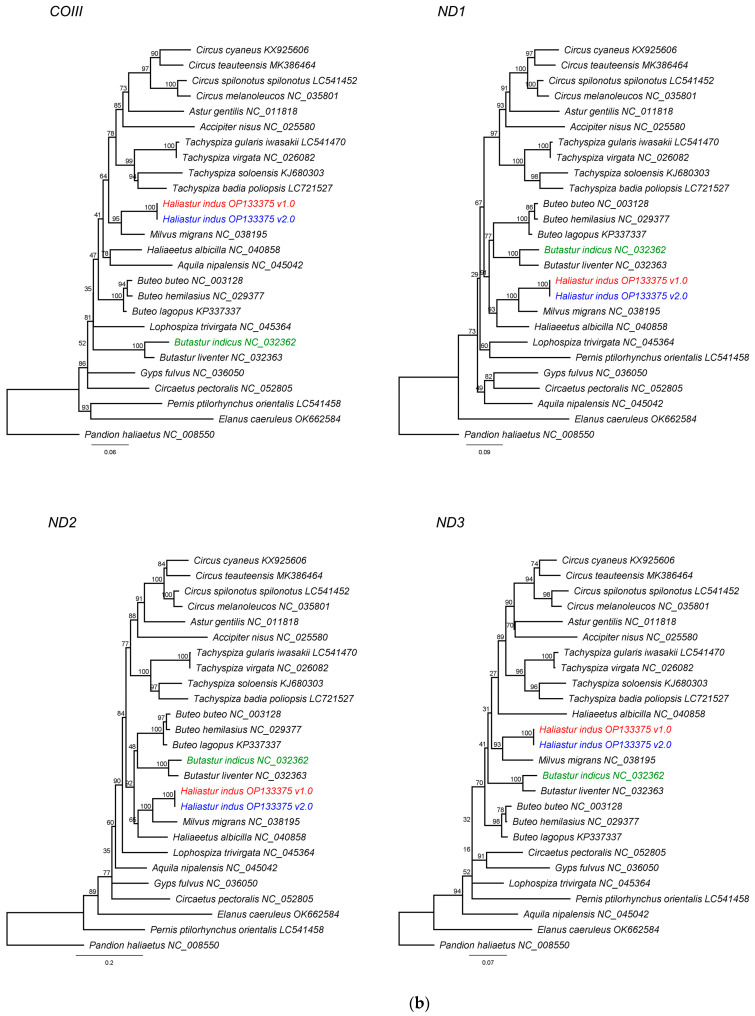

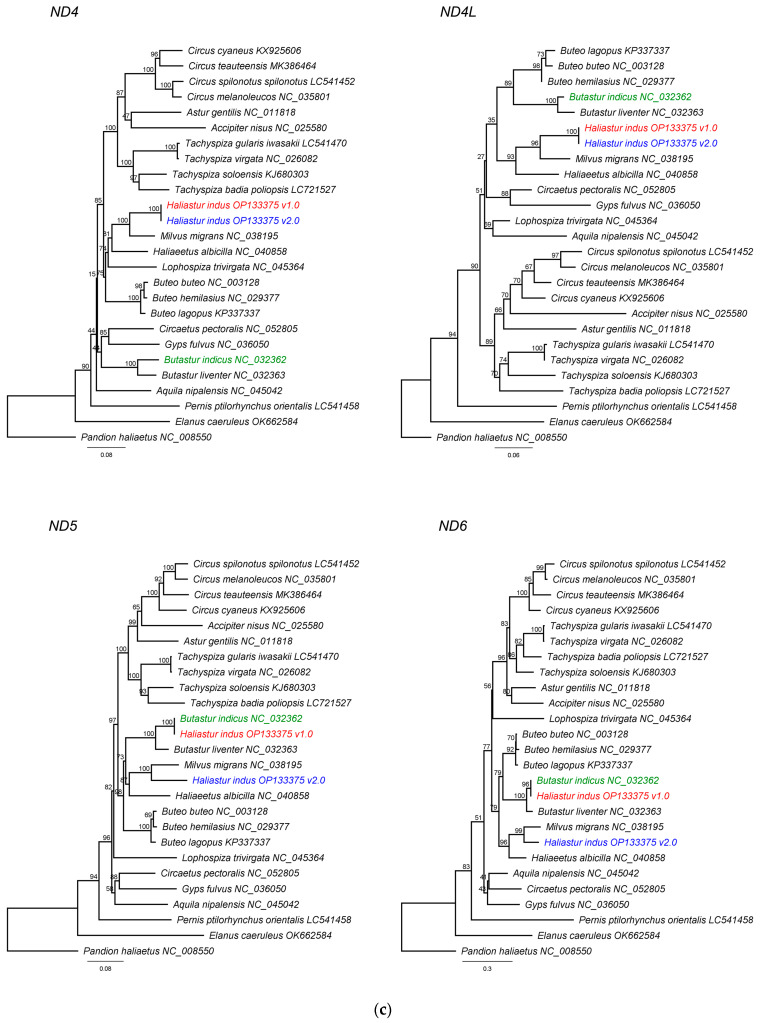

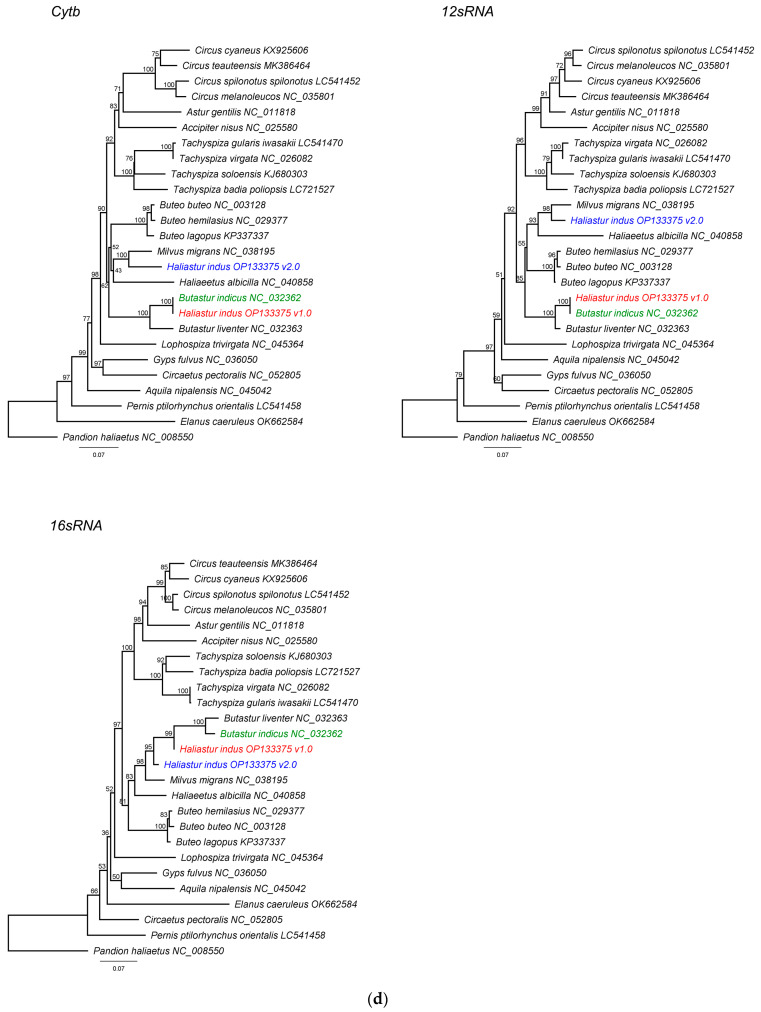
Maximum likelihood phylogenies of mitochondrial genes in Accipitridae, with *Pandion haliaetus* as outgroup (**a**) *ATP6*, *ATP8*, *COI*, and *COII*; (**b**) *COIII*, *ND1*, *ND2*, and *ND3*; (**c**) *ND4*, *ND4L*, *ND5*, and *ND6*; (**d**) *Cytb*, *12S rRNA* and *16S rRNA*. Green branches indicate the *Butastur indicus* reference mitogenome (NC_032362.1), red branches indicate the chimeric *Haliastur indus* mitogenome (OP133375.1), and blue branches indicate the corrected *H. indus* mitogenome (OP133375.2).
